# Laser ablation inductively coupled plasma mass spectrometry imaging of metals in experimental and clinical Wilson's disease

**DOI:** 10.1111/jcmm.12497

**Published:** 2015-02-20

**Authors:** Sorina Georgiana Boaru, Uta Merle, Ricarda Uerlings, Astrid Zimmermann, Christa Flechtenmacher, Claudia Willheim, Elisabeth Eder, Peter Ferenci, Wolfgang Stremmel, Ralf Weiskirchen

**Affiliations:** a Institute of Molecular Pathobiochemistry, Experimental Gene Therapie and Clinical Chemistry, RWTH Aachen University Hospital AachenAachen, Germany; b Department of Gastroenterology, Internal Medicine IV, University Hospital HeidelbergHeidelberg, Germany; c Central Institute of Engineering, Electronic und Analytics (ZEA-3), Research Centre Jülich (FZJ)Jülich, Germany; d Department of Pathology, University Hospital HeidelbergHeidelberg, Germany; e Division of Gastroenterology and Hepatology, Department of Internal Medicine III, Medical University of ViennaVienna, Austria

**Keywords:** metal bio-imaging, laser ablation inductively coupled plasma mass spectrometry, liver, trace metals, Wilson's disease

## Abstract

Wilson's disease is an autosomal recessive disorder in which the liver does not properly release copper into bile, resulting in prominent copper accumulation in various tissues. Affected patients suffer from hepatic disorders and severe neurological defects. Experimental studies in mutant mice in which the copper-transporting ATPase gene (*Atp7b*) is disrupted revealed a drastic, time-dependent accumulation of hepatic copper that is accompanied by formation of regenerative nodes resembling cirrhosis. Therefore, these mice represent an excellent exploratory model for Wilson's disease. However, the precise time course in hepatic copper accumulation and its impact on other trace metals within the liver is yet poorly understood. We have recently established novel laser ablation inductively coupled plasma mass spectrometry protocols allowing quantitative metal imaging in human and murine liver tissue with high sensitivity, spatial resolution, specificity and quantification ability. By use of these techniques, we here aimed to comparatively analyse hepatic metal content in wild-type and *Atp7b* deficient mice during ageing. We demonstrate that the age-dependent accumulation of hepatic copper is strictly associated with a simultaneous increase in iron and zinc, while the intrahepatic concentration and distribution of other metals or metalloids is not affected. The same findings were obtained in well-defined human liver samples that were obtained from patients suffering from Wilson's disease. We conclude that in Wilson's disease the imbalances of hepatic copper during ageing are closely correlated with alterations in intrahepatic iron and zinc content.

## Introduction

Progressive hepatolenticular degeneration, or Wilson's disease (WD), is a rare autosomal recessive disorder of copper metabolism which is characterized by hepatic and neurological abnormalities 1,2. The frequency of this malady is estimated to be between one in 30,000 and one in 100,000 individuals 1. The causative gene that is responsible for WD, ATP7B (OMIM 277900), is located on human chromosome 13 and encodes for a transmembrane protein ATPase that is highly expressed in the liver, kidney and placenta 3–5. It has dual synthetic and excretory roles by transporting copper into the trans-Golgi compartment and incorporating it into the plasma protein cerulopasmin and bile thereby controlling excretion of excess stores 1. There are several hundred different mutations within the *ATP7B* gene identified and most patients that suffer from WD are compound heterozygotes. Therefore, the penetrance of the disease and the clinical manifestations of WD are highly variable. Most prominent are hepatic alterations that are marked by persistently elevated activities of serum aminotransferases, inflammation, cirrhosis and fulminant hepatic failure that regularly necessitate liver transplantation. Neurological symptoms associated with WD are movement disorders, dystonia, insomnia and seizures. Additionally, psychiatric manifestations such as depression, neurotic behaviours, psychosis and personality changes are frequently observed in WD patients 6. Moreover, the formation of ocular Kayser–Fleischer rings (brownish-yellow rings appearing to encircle the iris) is a typical clinical sign of WD, especially in patients that develop neurological symptoms 7. However, there is presently no universal reliable test for diagnosis of WD and the symptoms of *ATP7B* mutations are often non-specific. Detailed formal guidelines for diagnosis and management of patient with WD that are mainly based on serum measurements (aminotransferases, ceruloplasmin), hepatic copper content and urinary excretion of copper were established by the American Association for the Study of Liver Diseases already some years ago 8,9 and more recently by the European Association for the Study of the Liver 10. Beside traditional tests and measurement of suitable biomarkers (serum ceruloplasmin, serum or urine copper levels), high-throughput genetic screening for WD mutations is becoming more widely available. However, some individuals still need a liver biopsy for hepatic copper quantification to finally confirm the diagnosis 1. Although *ATP7B* sequencing should be standard practice in the diagnosis of WD, these tests have also limitations and pitfalls mainly because the *ATP7B* gene is large and contains 21 individual exons with a coding region of 4.3 kb distributed over 80 kb of genomic DNA 4,5,11.

It is well known that the measurement of hepatic copper content alone allows only a rough estimation of the disease outcome because it is already known that other metals such as zinc may interfere with the toxicity of excess copper 12,13. Therefore, methods that allow simultaneous quantification of various metals should be highly advantageous to estimate the actual danger of hepatic copper overload and to predict disease progression.

We have recently established novel quantitative metal imaging protocols that are based on laser ablation inductively coupled plasma mass spectrometry (LA-ICP-MS). This method allows to image, measure and quantify trace metal distribution in cryo-sections of mouse and human liver tissue samples 14,15. We now extended these studies and performed comparative metal bio-imaging in wild-type and *Atp7b* deficient mice representing a well-accepted animal model resembling WD in humans 16–18. Our measurements confirm the gradual accumulation of hepatic copper during ageing that was already previously reported and that is correlated with the formation of tumorigenic lesions. Most interestingly, we found that the incremental copper accumulation is further associated with a simultaneous increase in hepatic iron and zinc, while the intrahepatic concentrations of other metals are not altered. Comparative analysis of liver specimens taken from normal and sequence verified WD patients confirmed these experimental findings suggesting that copper overload results in a complex change in hepatic trace element composition.

## Material and methods

### Animals

The establishment of *Atp7b* deficient mice was previously described 16. All mice and respective controls used in this study had a genetic 129/Sv background and were housed at the University of Heidelberg, according to the guidelines of the Institutional Animal Care and Use Committees and in accordance with governmental requirements 17. For our study, we analysed a total of 16 animals (controls: *n* = 8; *Atp7b*^−/−^: *n* = 8) at ages between 11 and 24 months. Moreover, we used several younger animals of 15 weeks for additional histochemical analysis of livers.

### Human samples

All human liver samples (controls: *n* = 4; WD samples: *n* = 6) were obtained from the central RWTH biomaterial bank (cBMB-UKA, http://www.cbmb.rwth-aachen.de/) or the University Hospital of Heidelberg. Patients' characteristics are given in Table S1. Each patient from whom material was analysed provided written informed consent and the study was approved by the local ethics committees at the RWTH Aachen University and the University Hospital Heidelberg. The occurrence of *ATP7B* mutations was confirmed by direct sequencing or alternatively direct mutation detection by using allele-specific probes 19.

### RNA isolation and quantitative real time PCR

RNA isolation from murine or human liver tissues was done essentially as described before 20. Total RNA was quantified and 2 μg samples each were reverse transcribed using the Superscript II reverse transcriptase and random hexamer primers (both from Invitrogen, Life Technologies, Darmstadt, Germany). For the individual TaqMan PCR assays, the cDNA derived from 25 ng RNA was amplified in a total volume of 25 μl using the qPCR Core Kit (Eurogentec, Cologne, Germany) and primer combinations listed in Table S2. The amplification of all murine (IL-1β, TNF-α, NLRP3, ASC, TIMP-1, MMP-9 and GAPDH) or human (IL-1β, NLRP3, ATP7b, caspase-1, TNF-α, ASC, TIMP-1, MMP-9 and GAPDH) target gene sequences was done under the following conditions: initial melting phase at 95°C for 10 min., followed by 40 cycles at 95°C for 15 sec. and 60°C for 1 min. respectively. All samples were normalized to the expression of *GAPDH* measured in the same sample.

### Western blot analysis

Protein extracts from human and murine livers were prepared following standard protocols 21. Equal amounts of proteins (100 μg) were heated at 80°C for 10 min. and separated in 4–12% Bis-Tris gels (Invitrogen) under reducing conditions. Proteins were then electro-blotted on nitrocellulose membranes (Schleicher & Schuell, Dassel, Germany). Successful protein transfer and equal protein loading was monitored by Ponceau S staining. Unspecific binding sites were blocked in TBST [10 mM Tris/HCl, 150 mM NaCl, 0.1% (v/v) Tween 20 (pH 7.6)] containing 5% (w/v) non-fat milk powder. The membranes were probed with antibodies given in Table S3. Primary antibodies were detected with horseradish-peroxidase-conjugated secondary antibodies (Santa Cruz, Santa Cruz, CA, USA) and the Supersignal™ chemiluminescent substrate (Perbio Science, Bonn, Germany). The chemiluminescent signals were visualized in a Lumi-imager (Roche Diagnostics, Mannheim, Germany).

### Histological evaluation of human liver sections

Haematoxylin and eosin, Masson-Goldner trichrome and copper stains were done using commercially available kit staining kits following standard protocols.

### LA-ICP-MS imaging of trace metals in murine and human liver tissue sections

In the experimental setup that we used for our measurements, a quadrupole-based inductively coupled plasma mass spectrometer (XSeries 2, Thermo Scientific, Bremen, Germany) was coupled to a laser ablation system (NWR 213; New Wave Research, Fremont, CA, USA). For metal imaging in murine and human liver tissue, 30 μm thick tissue cryo-sections were prepared and laser ablation of biological tissue was performed essentially under conditions that were described before 14. The ablated material was transported by argon gas into the inductively coupled plasma and the formed ions were extracted in the ultrahigh vacuum mass spectrometer *via* a differential pumped interface, separated in the quadrupole mass analyser according to their mass-to-charge ratios, and detected by an ion detector (Fig. S1). All trace metal concentrations were calculated from ion intensities averaged throughout freely drawn regions of interest and representative images were generated from the continuous list of raw pixel values using modified in-house LA-ICP-MS Image Generation software that is based on the IMAGENA software originally created at the Research Centre Jülich 22. For quantification purposes, matrix-matched laboratory standards were prepared by dosing each analysed element to the pieces of homogenized tissue that were essentially prepared as described 15. As a surrogate of slice thickness, the metal intensities were normalized to the average ^13^C ion intensity of respective samples in each measurement as suggested elsewhere 23.

## Results

First we evaluated the liver histology in animals lacking a functional Atp7b transporter. Therefore, we stained histological specimens of respective animals at age 15 weeks either with haematoxylin and eosin or Sirius Red (Fig. S2). Compared to age-matched controls, the *Atp7b* deficient mice showed hypertrophic hepatocytes and first signs of inflammation and hepatic fibrosis that were further documented in elevated mRNA expression of inflammatory marker IL-1β and TNF-α as well as TIMP-1, MMP-9 and the inflammasome components NLRP3 and ASC (Fig. S3A). Likewise, most of the livers taken from the *Atp7b* deficient mice showed increased α-smooth muscle actin (α-SMA) and TIMP-1 protein expression (Fig. S3B).

**Fig 1:**
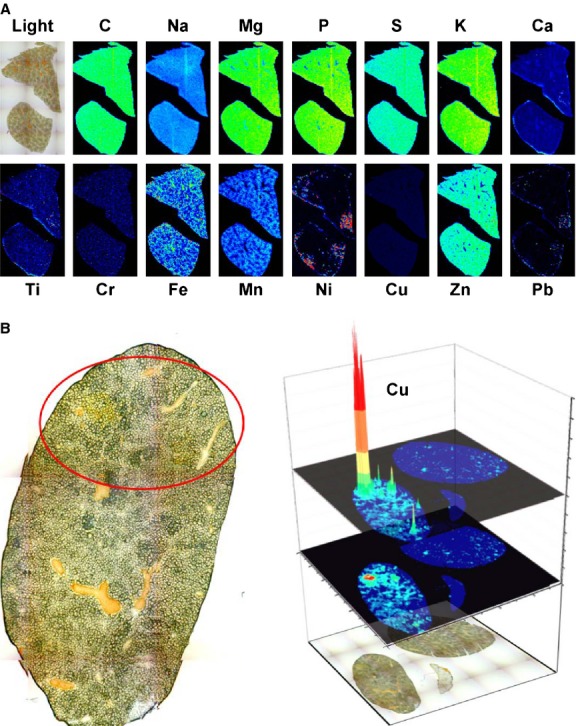
Biometal imaging in liver. (A) Simultaneous LA-ICP-MS imaging of C, Na, Mg, P, S, K, Ca, Ti, Cr, Fe, Mn, Ni, Cu, Zn, and Pb isotopes in a liver section taken from a 10 months old wild-type animal. (B) A liver section of a 11 months old male mouse lacking the copper transporter gene *Atp7b* that had already developed a tumour (*left panel*) was analysed for its copper content by LA-ICP-MS imaging (*right panel*). In the respective analysis, the distribution of Cu is depicted as a three-dimensional representation.

Next we generated 30 μm thick cryosections and measured the distributions of various metals by LA-ICP-MS in sections that were obtained from wild-type control mice (Fig.1A) showing that most of the imaged metals and metalloids had a homogenous distribution within the normal tissue with the exceptions of iron and manganese that were following a defined, uneven distribution pattern. The homogenous distribution of carbon and sulphur indicates that the thickness of our sections were the same everywhere, prompting us to use these elements as internal standards for section thickness to which all metal measurements can be normalized. In the normal tissue, we found only low concentrations of copper (see also below) with a homogenous distribution throughout the complete tissue.

In older homozygous null mice we could confirm the typical morphological abnormalities in liver pathology resembling cirrhosis (Fig.1B, *left panel* and not shown) that were reported before 16. Most likely these pathogenetic alterations represent the consequence of chronic inflammation and mild fibrosis that were already noticed in younger animals (cf. Fig. S2).

When we imaged the hepatic copper concentration in a 10 months old animal lacking *Atp7b,* we found an irregular copper distribution with extremely high regional concentrations (Fig.1B, *right panel*).

**Fig 2:**
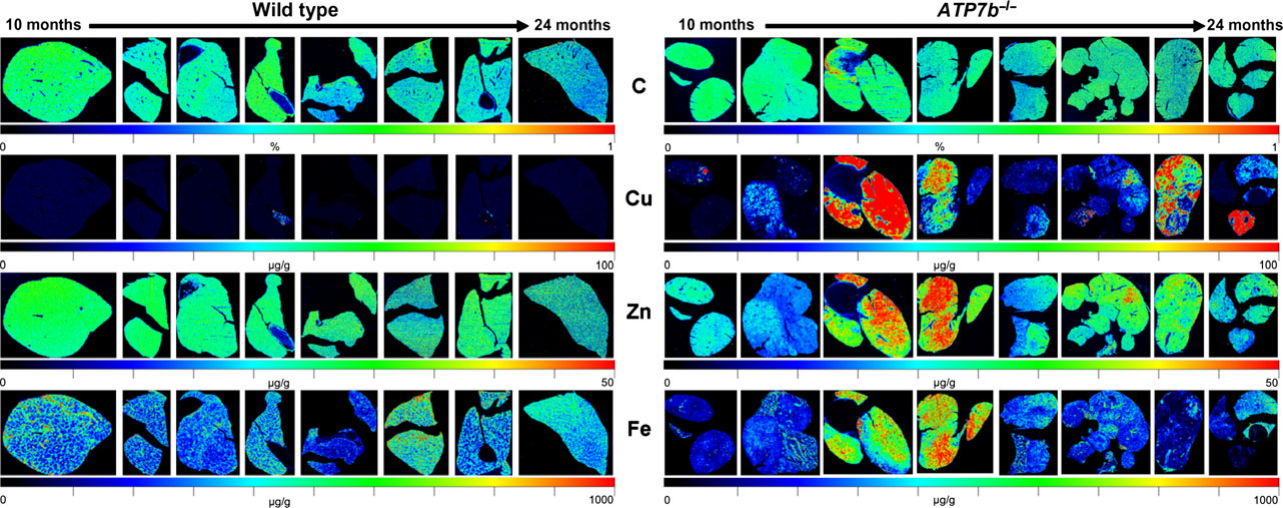
Accumulation of copper in *Atp7b*^−/−^ mice as demonstrated by LA-ICP-MS imaging. 30-μm thick cryo-sections from wild-type (*left panel*) and *Atp7b*^−/−^ mice (*right panel*) at age between 10 and 24 months were subjected to LA-ICP-MS imaging and the content of carbon (C), copper (Cu), zinc (Zn), and iron (Fe) determined. Please note that the content of C that served as reference is given in %, while the total concentrations of Cu, Zn, and Fe are given in μg/g liver tissue.

When we next analysed the metal content in mice at different ages, we found progressive hepatic copper accumulation in *Atp7b* deficient mice with highest intrahepatic copper concentrations in animals at age of 14 months, while the hepatic copper concentration stayed constantly low in wild-type control animals during the complete life span (Fig.2). Interestingly, we determined that the intrahepatic accumulation of copper in the *Atp7b* deficient mice was linked with a simultaneous accumulation of zinc and iron (Fig.2). This phenomenon was particularly visible in animals at an age of 1 year (Fig. S4).

**Fig 3:**
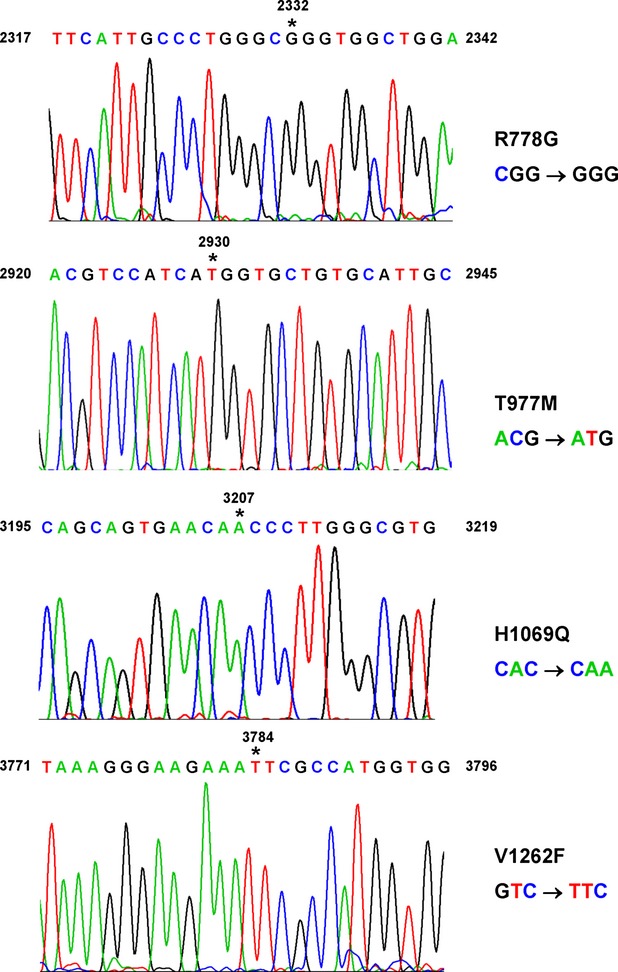
Sequence analysis of mutations underlying Wilson's disease. DNA from WD patients were isolated and sequenced by standard procedures. Individual mutations sites are indicated by asterisks. The numbering of nucleotides is given according to GenBank entry with accession no. U03464.1 starting with the translational start codon (ATG) at position +1. The nucleotide substitutions at position +2332, +2930, +3207, and +3784 result in nonsynonymous amino acid exchanges at position 778 (R778G), 977 (T977M), 1069 (H1069Q) and 1262 (V1262F) respectively.

We next wanted to test if the observed simultaneous alterations in copper, zinc and iron were also detectable in specimens obtained from resected livers that were derived from well-characterized WD patients in which the clinical diagnosis ‘WD’ was also confirmed by verification of disease-causing *ATP7B* gene mutations (Fig.3). Compared to normal human control liver samples that showed a balanced expression of ATP7B, TIMP-1, MMP-9, NLRP3, IL-1β, ASC, Caspase 1 and TNF-α (Fig. S5A), the expression of these genes was more variable in the diseased livers as confirmed by quantitative real time PCR and Western blot analysis (Fig. S5B and C). Typically, the livers of respective patients showed some fat containing hepatocytes, portal and periportal inflammation and bilirubinostasis (Fig. S6).

**Fig 4:**
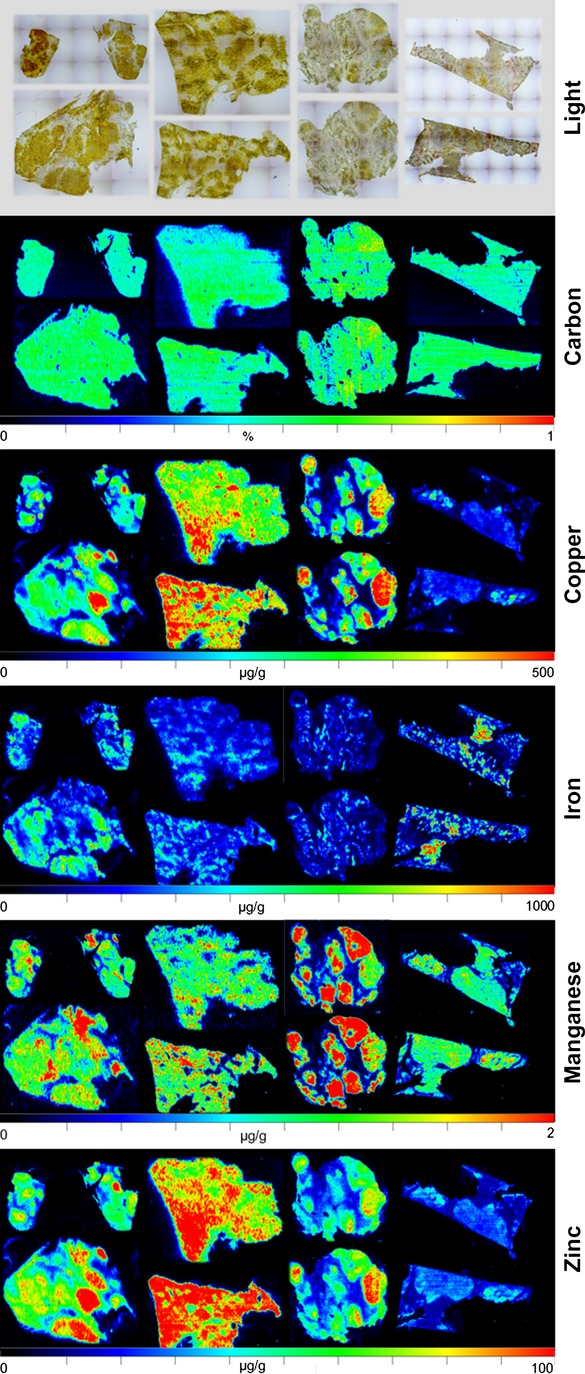
Metal bio-imaging in human samples taken from patients with Wilson's disease. Representative LA-ICP-MS imaging maps of C, Cu, Fe, Mn and Zn are given. In the upper panel, microphotographs of the analysed samples are shown.

When we analysed the hepatic metal content in control samples by LA-ICP-MS, we found that the copper concentration was lower than 50 μg/g tissue in all control tissues, while iron and zinc showed individual variances with concentrations ranging from 100 to 400 μg/g and 30 to 70 μg/g liver tissue respectively (Fig. S7). In contrast, the liver tissues from WD patients had regions with massive elevated copper concentrations that reached regional values over 500 μg/g liver tissue (Fig.4). Similar to the observations that we made in the *Atp7b* deficient mice, the iron and zinc concentrations were simultaneously elevated in the WD human livers in regions that showed high copper content. Interestingly, this analysis further revealed that in humans the correlation of copper is highest with zinc and that the concentration of manganese is inversely correlated with those of copper in the diseased human samples.

## Discussion

The spectrum of liver disease and the clinical features encountered in patients with WD can be highly variable ranging from persistent asymptomatic hepatomegaly or elevation of serum aminotransferases to acute liver failure 9,10. Noteworthy, the copper serum concentration in WD patients is usually decreased in proportion to the decreased ceruloplasmin in the circulation 9. Even in patients with severe liver injury, serum copper concentrations are in the normal range, while the concentration might increase abruptly in the setting of acute liver failure because of the sudden release of hepatic copper 9. Presently, there is an intensive debate about the hepatic parenchymal copper concentration that is indicative for WD and a threshold value equal or higher of 70–250 μg/g dry weight of liver is assumed to display the occurrence of WD 7,24. One of the major problems in determination of hepatic parenchymal copper concentration is the fact that this element is distributed inhomogeneously within the tissue during later stages of WD resulting in sampling error and underestimation of total copper concentration 9. Therefore, the liver copper content in diagnosis was already critically challenged decades ago 25. Moreover, it was recognized in 1979 that oral zinc sulphate prevents storage of copper in the liver and contributes to the mobilization and excretion of copper deposits 26. Later studies revealed that zinc supplementation induces metallothionein in the hepatocyte that has favourable effects and interferes with the toxicity of copper 27,28.

All these findings indicate that there is (*i*) an urgent need in upgrading hepatic copper measurements in regard with diagnostic sensitivity, spatial resolution, specificity and quantification ability and that (*ii*) the simultaneous measurement of zinc and potentially other metals would add a novel amendment to the actual diagnosis and management of WD.

We have recently established a quantitative biometal imaging technique of mouse and human liver tissue allowing simultaneous measurement and quantification of various metals in thin cryo-sections 14,15. This methodology is based on LA-ICP-MS which is capable to detect metals and several non-metals at very low concentrations with minimal requirements in sample preparation and processing expenditures 29.

In this study, we asked if this methodology is suitable to determine the reported age-dependent accumulation of copper in an experimental model of WD in which the *Atp7b* gene is disrupted. Histological analysis of respective livers of *Atp7b* deficient mice revealed the expected occurrence of hypertrophic hepatocytes. In addition, the progression of parenchymal damage, inflammation and fibrosis in these livers from respective animals was noticeable in Sirius Red stains and further indicated by elevated mRNA and protein expression of the inflammation- and fibrosis-associated genes IL-1β, TNF-α, NLRP3, ASC, TIMP-1 and MMP-9.

To demonstrate the hepatic distribution of various metals and metalloids in normal control mice, we generated cryosections of respective livers and performed LA-ICP-MS imaging following our protocols that we have published before 14,15. This analysis revealed that Na, Mg, P, K, Ca, Ti, Cr, Ni, Cu, Zn and Pb showed a homogenous distribution with the tissue. In our analysis, the regional measurement of C and S were taken for internal standardization and confirmation of equal section thickness as suggested elsewhere 23. In the control sections, the distribution of Fe showed minor variations with decreasing gradients from periportal to centrilobular areas. When we imaged liver sections taken from an 11 month-old *Atp7b* deficient mouse that had already developed hepatic tumours, we found that the concentration of copper was extremely high in regions of tumorigenic nodules, possibly suggesting a direct correlation of copper concentration, cell/tissue damage, and tumour formation. Consistent with this assumption, a previous study that analysed the effects of copper overload on the survival of human hepatoma cell line HepG2 has shown that copper induces in a dose-dependent manner reactive oxygen species production and other deleterious effects 30.

In our analysis, we found that copper accumulates throughout the liver tissue of *Atp7b* deficient mice during the life span with highest intrahepatic levels at 14 months. Thereafter, the intrahepatic copper concentration decreases, possibly indicating the ongoing tissue damage that is associated with intrahepatic copper loss in these animals at higher age 16. We found that at later stages, the concentration of copper not only decreases but further shows an inhomogeneous distribution within the organ. Interestingly, when we determined the concentration of zinc and iron in affected animals, we noticed a simultaneous accumulation of both metals in affected organs, while the concentration of both metals was not altered during the prolonged life span in control mice. The gradual increase in both total hepatic copper and iron in animals between 3 and 47 weeks was already reported before 17. However, the respective quantification was done in liver extracts using atomic absorption that provided no further information about the regional distribution of both metals in the tissue. We here demonstrate that the accumulation of the different metals is combined. The mutual increase in copper, iron and zinc was particularity noticeable in animals at an age of 1 year, while the different metal concentrations were not necessarily coupled at higher ages potentially again reflecting ongoing hepatic insult that is characterized by parenchymal cell necrosis and decline in organ integrity. This assumption was underpinned when we imaged copper, iron, and zinc at higher resolution showing that all three metals decreased in regions in which nodules had already developed.

In our study, we observed similar alterations in metal deposits in human livers of WD patients that showed the typical disease-associated clinical features. The respective missense mutations within the enrolled patients affected amino acids 778 (R778G), 977 (T977M), 1069 (H1069Q) or 1262 (V1262F). These mutations are all associated with a significantly reduced copper ATPase expression or activity resulting in lower or absence of copper export capacity 31–34. Compared to normal human control liver samples that showed a relatively balanced expression of ATP7B, TIMP-1, MMP-9, NLRP3, IL-1β, ASC, Caspase 1 and TNF-α that was reflected in small standard derivations, the relative mRNA abundance of respective genes was more variable in the WD liver samples. Moreover, the samples showed a clear indication of ongoing hepatic fibrosis as indicated by elevated levels of TIMP-1 and MMP-9, markers that indicate inflammatory response (IL-1β and TNF-α) and histological findings that are characteristic for WD livers including hyperplasia, cirrhosis and abundant deposits of copper and extracellular matrix components.

In the normal control samples, the copper content within the tissue was overall lower than 50 μg/g tissue, iron ranged from 100 to 400 μg/g tissue, while the zinc concentration was between 30 and 70 μg/g liver tissue. In sharp contrast, the liver tissue from the analysed WD patients contained regions in which copper reached values over 500 μg/g liver tissue. Likewise to our finding in the experimental WD model, the iron and zinc concentrations were elevated in regions that showed higher copper concentrations.

Presently, we do not know if this finding is specific for our experimental model and the few patients that we have tested, but there are a large variety of experimental and clinical studies available that found correlation between intrahepatic copper, zinc and iron. In the toxic milk mouse model that represents another established experimental model for WD, hepatic zinc was specifically increased with age within the liver 35. Also iron accumulation in the liver of patients with WD was reported in four male patients without a background of hemochromatosis 36. Another older report described excess hepatic copper and zinc in patients with advanced biliary cirrhosis 37. It will be interesting to analyse in future work how this simultaneous metal imbalance in copper, zinc and iron occurs.

Interestingly, the regional distribution of manganese was highly inhomogeneous in the tested WD liver samples with highest concentrations of 2 μg/g tissue. As it is known that orally administered manganese is an effective and highly sensitive agent to detect liver metastasis 38,39, it is tempting to speculate that the regions in which manganese shows highest concentrations are already tumorigenic and that further the measurement of manganese is potentially an effective means to identify liver tissue that is prone for metastasis or hyperplasia. On the other hand it might be possible that the accumulation of manganese and zinc are secondary events. Under normal conditions, 95% of manganese is eliminated by biliary excretion that is reduced when liver damage occurs 40. This assumption is supported by findings showing that chronic liver failure leads to manganese intoxication 41. Manganese itself can participate in Fenton reactions and thus induce reactive oxygen formation and oxidative damage that is for example increased in human liver tissue adjacent to hepatocellular carcinoma 42. Likewise, zinc is transported into bile and it is therefore reasonable that the elevated concentrations of zinc in older animals and WD patients are the consequence of progressive liver damage. A good explanation for the observed iron accumulation detected in liver samples of both *Atp7b* deficient animals and WD patients is not immediately apparent. However, a similar finding was previously reported in a small cohort (*n* = 4) of male WD patients 36. The authors of that study suggested that copper toxicosis leads to hypoceruloplasminemia that predicts secondary iron overload. Although we have not tested if this hypothesis is correct, in a more recent study in which various copper and iron overload patterns were analysed in livers of WD patients and idiopathic copper toxicosis the connection of iron accumulation and hypoceruloplasminemia was strongly supported 43.

All these findings of our study indicate that the LA-ICP-MS technology is not only an elegant technique for simultaneous measurement of various metals and metalloids in biomedical research but may also be in the future a significant addition to the diagnostic toolkit for WD and any other hepatic disease that is initiated or modulated by metals, metalloids or combinations thereof.

**Fig 5:**
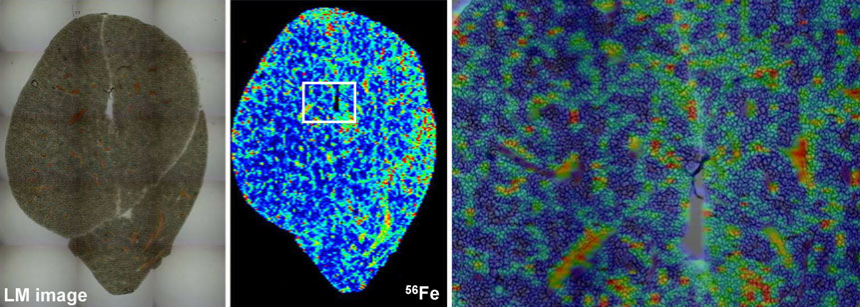
High resolution imaging of hepatic iron in wild-type mouse. The content of iron in a liver section of a wild-type animal at age of 10 months (cf. Fig.2, *left hand sided section*) was determined by LA-ICP-MS. The left panel shows a microphotograph of the section, while the image in the middle depicts an overview of iron in respective section. The white boxed area is shown at higher magnification in the right panel demonstrating that LA-ICP-MS imaging allows metal imaging at high resolution.

Recently, protocols were established allowing simultaneous elemental and molecular MS imaging that will open further new possibilities to address complex challenges in life science research and diagnostics 44. We are trying to optimize our metal imaging protocols in regard with spatial resolution that together with the measurement time required to image larger sampling areas somewhat restricts this versatile, high sensitivity technique 45. With our present protocols, we are able to provide metal imaging data with nearly cellular resolution (Fig.5). In future, we hope to improve the spatial resolution of our methods and develop hardware with more sensitive imaging capabilities allowing to measure and quantify metals at the subcellular level.

In summary, it is obvious that the current work has potential implications for the development of novel therapeutic strategies to improve clinical management of liver diseases that are associated with imbalances of metals such as WD and hemochromatosis. LA-ICP-MS has a high spatial resolution (10–60 μm spot size, 5–20 μm depth) and needs a low time for isotope analysis (2–3 min. per metal) combined with a high precision and accuracy (2–8% precision per analysis). Consequently, LA-ICP-MS can be performed with high sample throughput. A multitude of different metals, metalloids and non-metals can be measured and quantified simultaneously and the regional distribution of various metals determined in the same run. Furthermore, there is no need for pre-analytic processing and LA-ICP-MS has minimal sample requirements allowing accurate metal measurements in small specimens. Compared to traditional diagnosis that is mainly based on biomarkers that are indirectly linked to the concentration of a specific metal, LA-ICP-MS provides real-time information about the direct metal content within a particular sample.

In summary, metal imaging by LA-ICP-MS is a powerful method that is simple, more accurate, cheaper and faster than the currently used method of metal deposit diagnosis. It is potentially also advantageous in the diagnosis of acute and chronic metal intoxication. These frequently occur in the course of long-term parenteral nutrition, organ failure, medical treatments, cutaneous exposure and unwanted uptake though the digestive tract or the respiratory system.

## Supporting Information

**Figure S1** Laser ablation inductively coupled plasma mass spectrometry in hepatic metal imaging.

**Figure S2** Diagnostic evaluation of murine liver tissue in Haematoxylin and Eosin and Sirius Red stain.

**Figure S3** Gene and protein expression analysis in experimental Wilson's disease.

**Figure S4** High resolution imaging of copper, iron, and zinc in liver sections of age-matched wild-type and *Atp7b-*deficient mice.

**Figure S5** Gene expression and protein expression in normal and Wilson's disease liver samples.

**Figure S6** Histological analysis of liver specimen taken from control patients and Wilson's disease patients.

**Figure S7** Metal bio-imaging in human control liver samples.

**Table S1** Patient's characteristics.

**Table S2** Primers used in this study.

**Table S3** Antibodies used in this study.
