# Supplementary figures and images for: Laser ablation inductively coupled plasma mass spectrometry imaging of metals in experimental and clinical Wilson's disease
Source: J Cell Mol Med. 2015 Feb 20;19(4):806–14. doi: 10.1111/jcmm.12497 (PMC4395195; doi:10.1111/jcmm.12497)

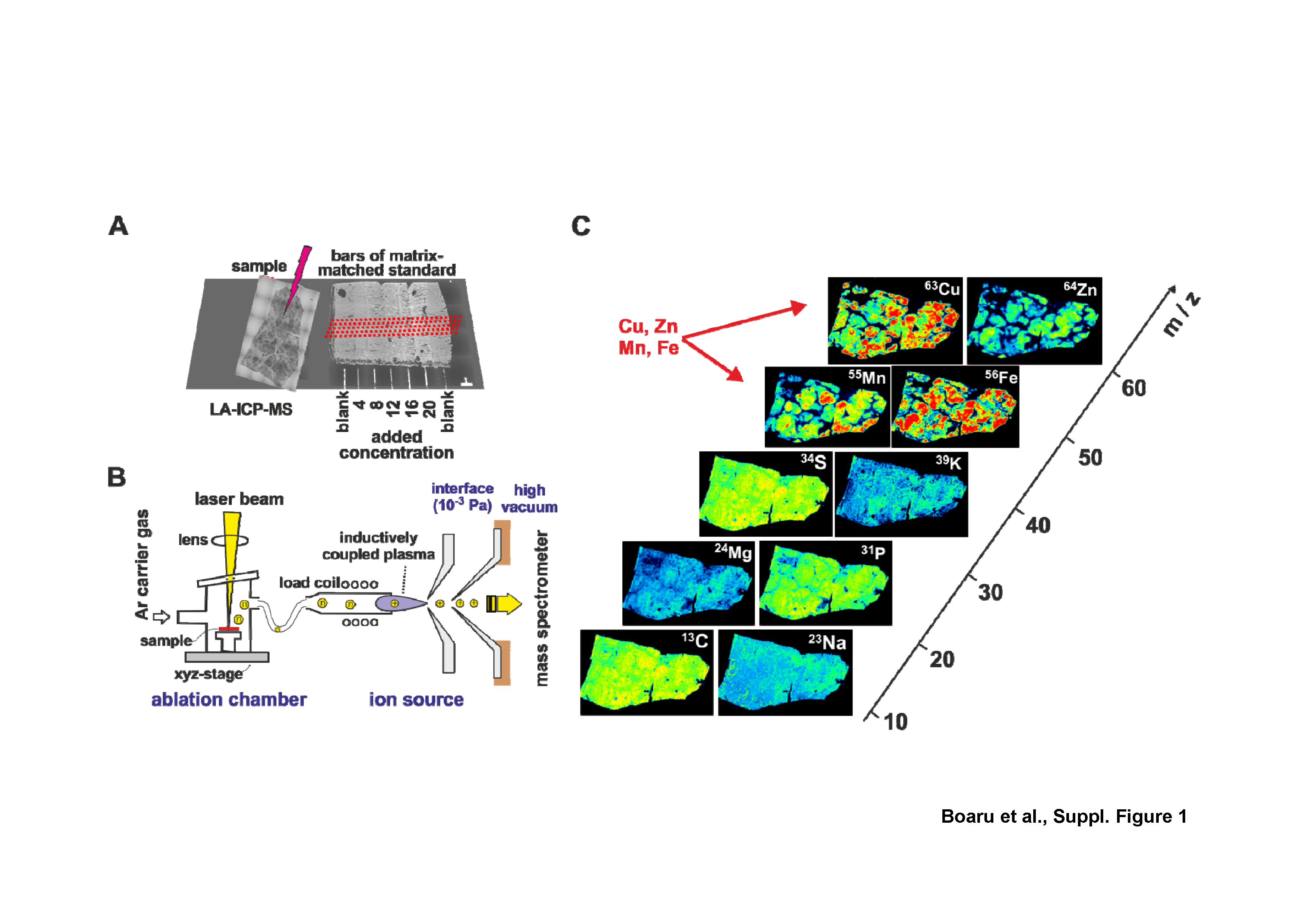

Supplement: Supplementary file 1 [file jcmm0019-0806-sd1.tif]

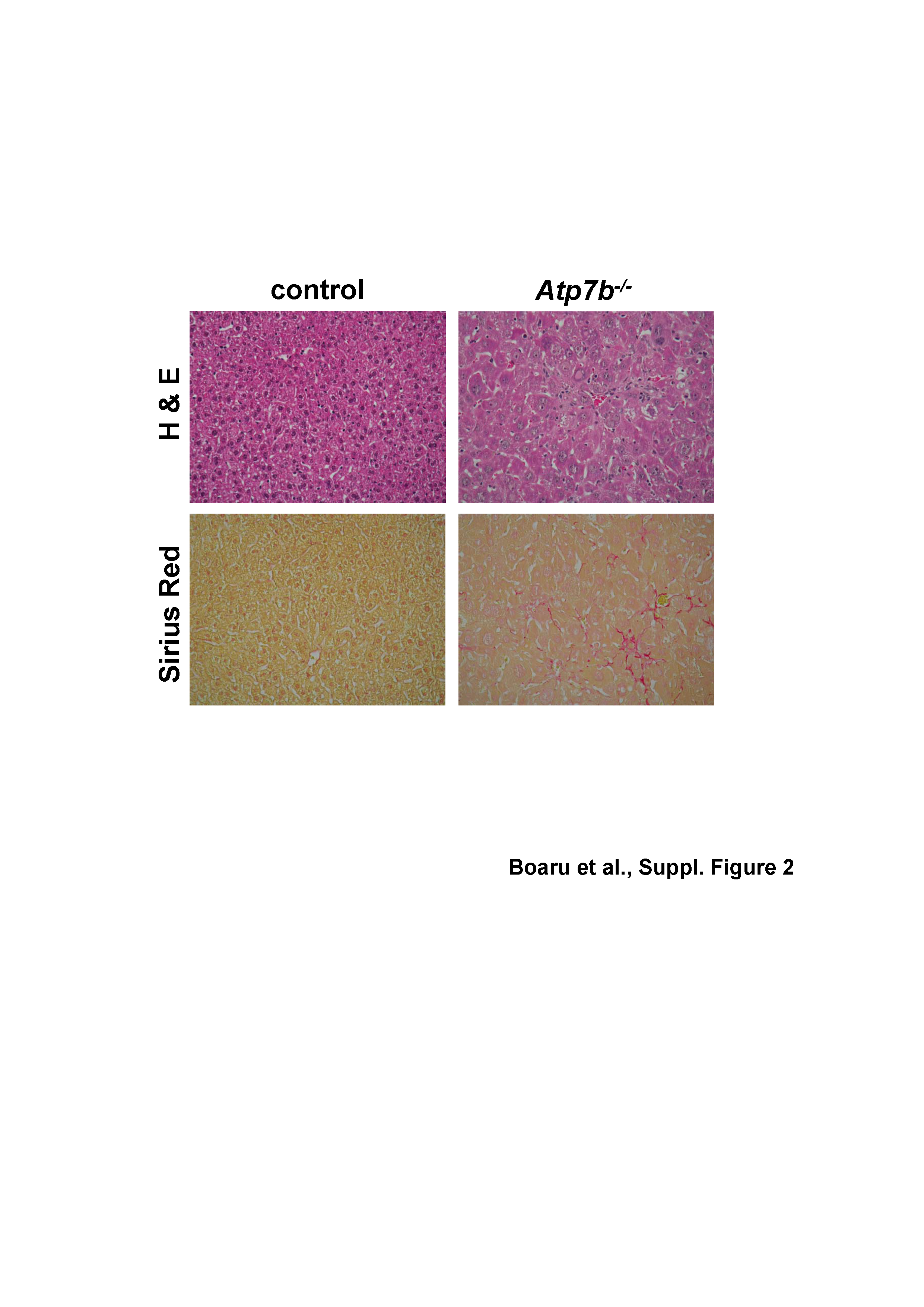

Supplement: Supplementary file 2 [file jcmm0019-0806-sd2.tif]

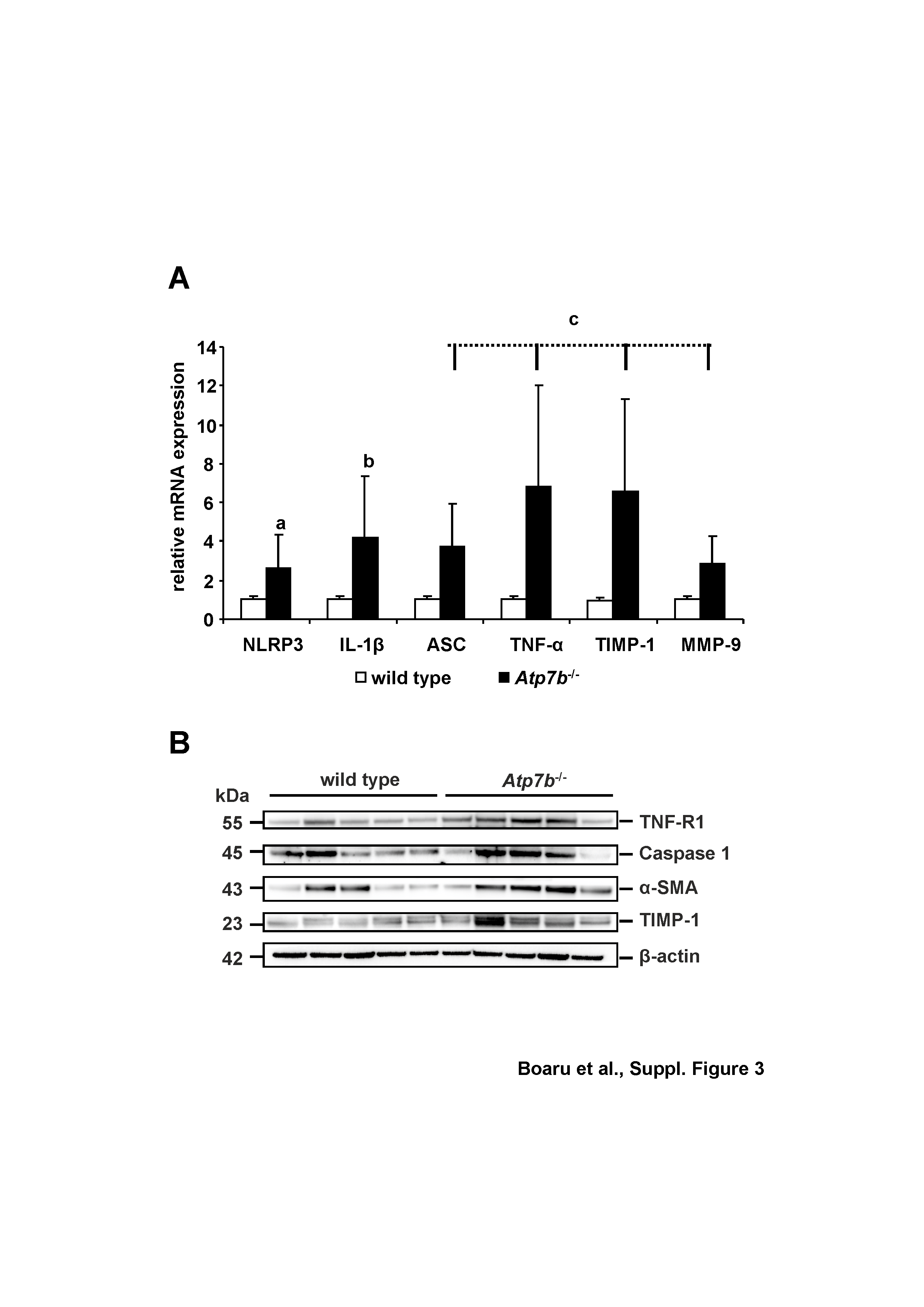

Supplement: Supplementary file 3 [file jcmm0019-0806-sd3.tif]

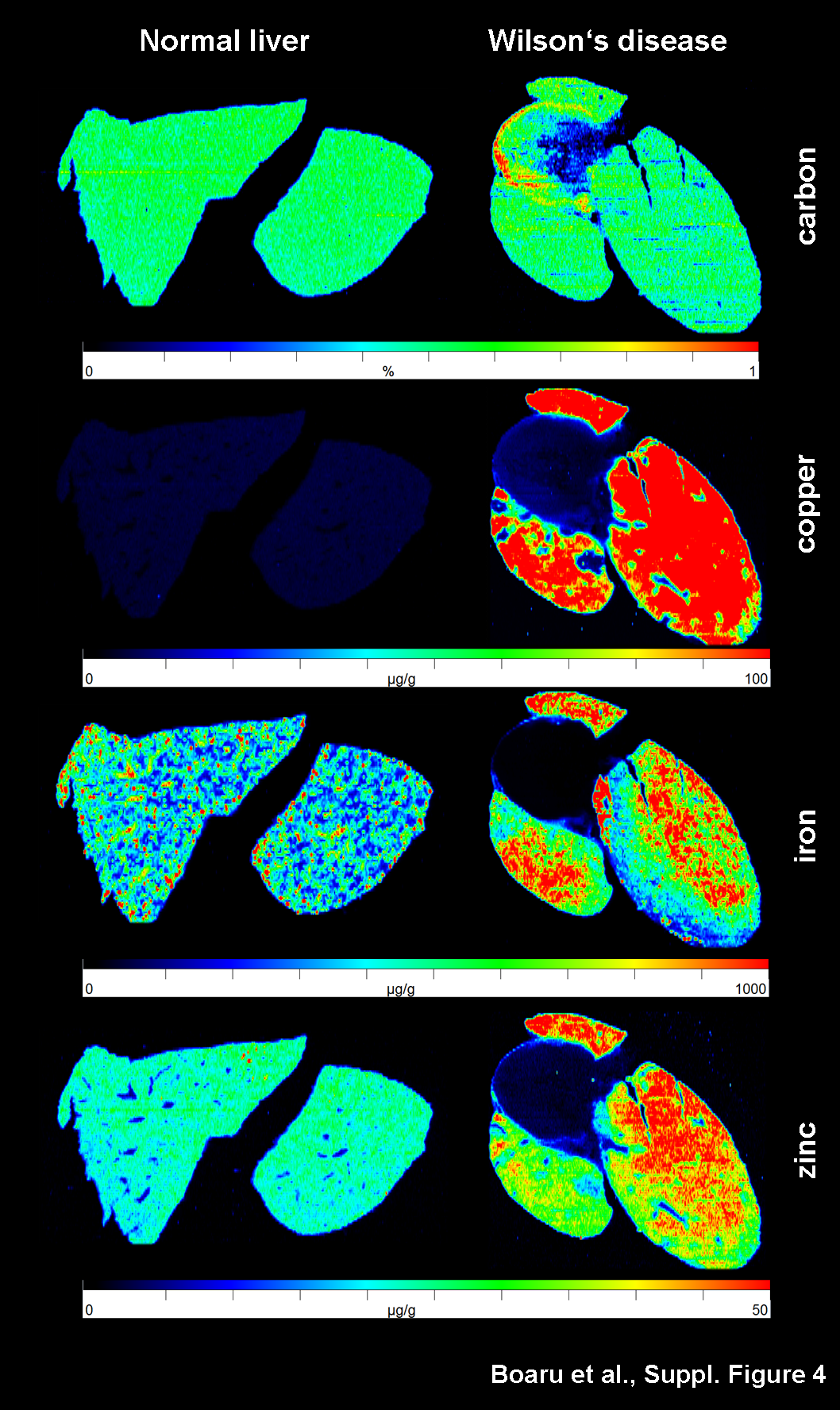

Supplement: Supplementary file 4 [file jcmm0019-0806-sd4.tif]

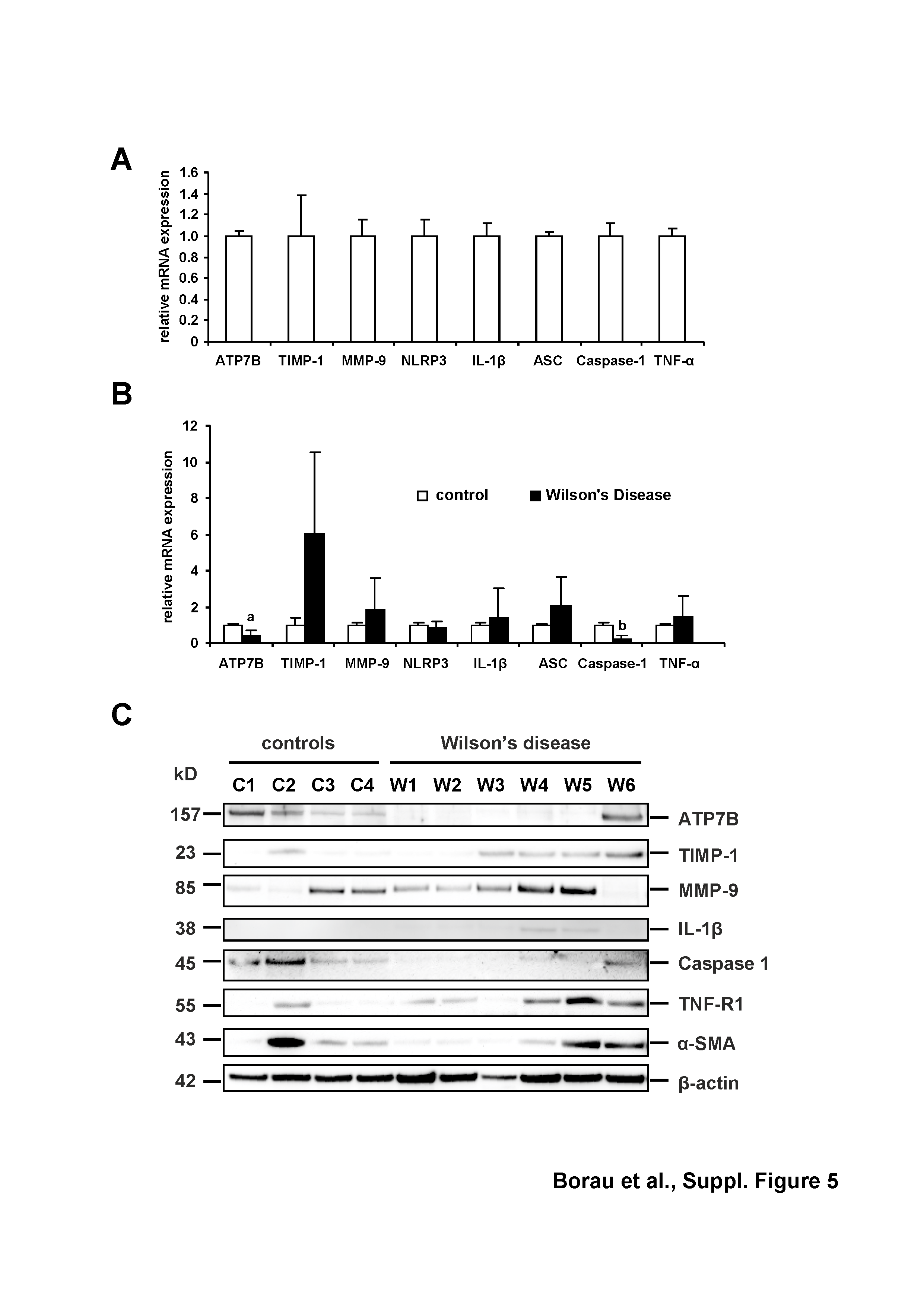

Supplement: Supplementary file 5 [file jcmm0019-0806-sd5.tif]

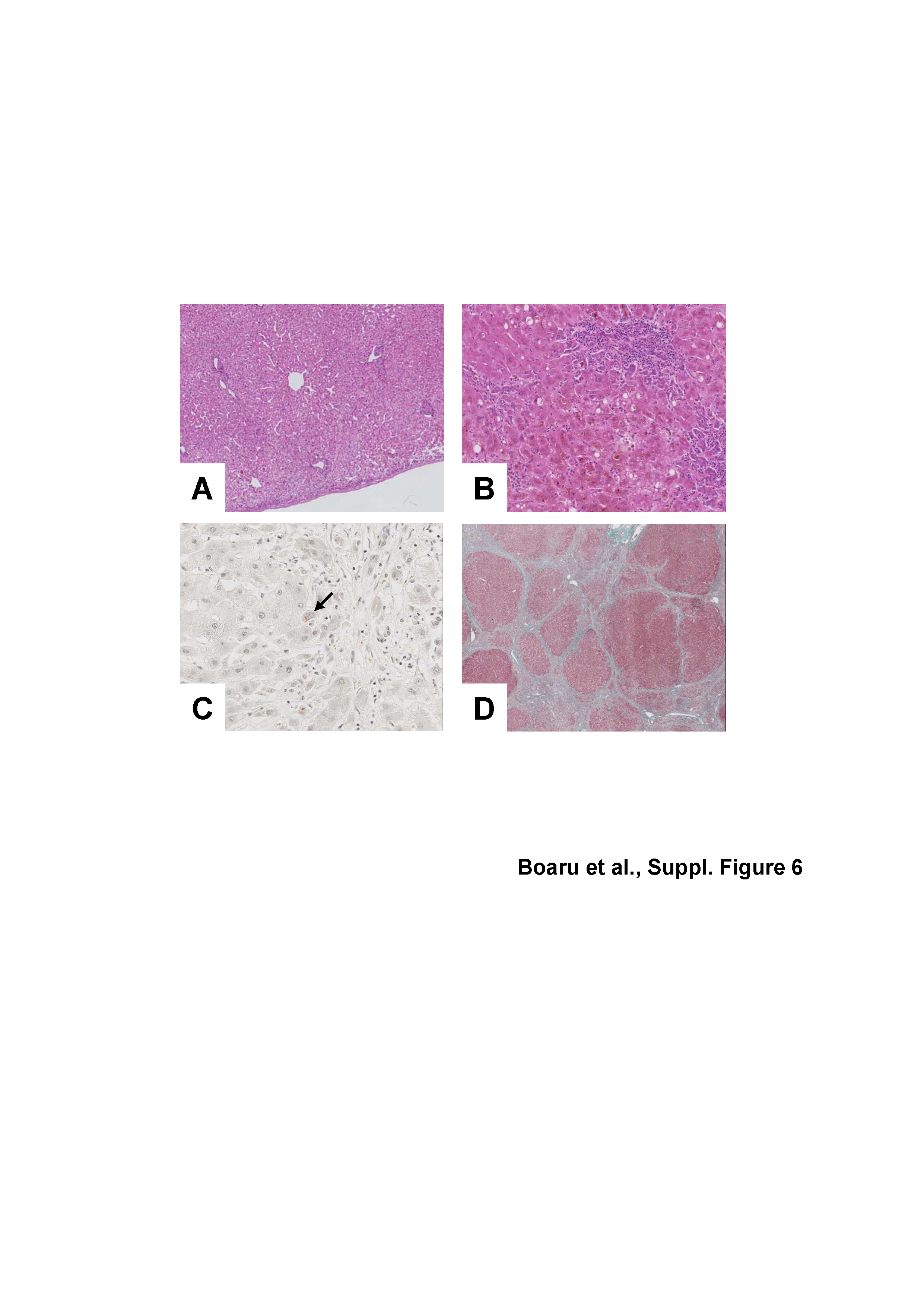

Supplement: Supplementary file 6 [file jcmm0019-0806-sd6.tif]

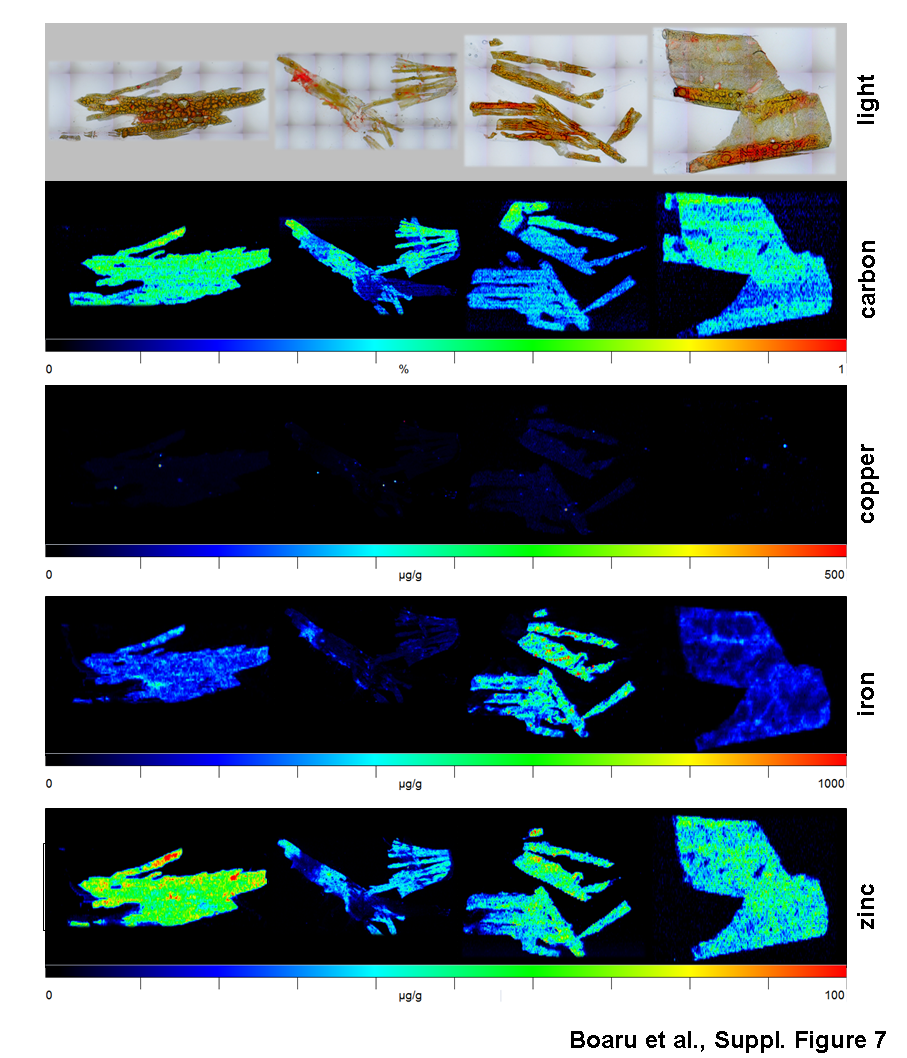

Supplement: Supplementary file 7 [file jcmm0019-0806-sd7.tif]
