# Supplementary material for: Laser ablation inductively coupled plasma mass spectrometry imaging of metals in experimental and clinical Wilson's disease
Source: J Cell Mol Med. 2015 Feb 20;19(4):806–14. doi: 10.1111/jcmm.12497 (PMC4395195; doi:10.1111/jcmm.12497)
Supplement: Supplementary file 8 [file jcmm0019-0806-sd8.doc]

**Supplementary Table 1**

**Patient'**s characteristics

| **Patient no.** | **Internal no.** | **Sex/Patient characteristics/** |
| --- | --- | --- |
| **C1** | JDZKND | m; normal liver histology |
| **C2** | OEUOMK | f; normal liver histology |
| **C3** | RUYSGC | m; normal liver histology |
| **C4** | RWQWFH | f; normal liver histology |
| **D1** | 1 | f; H1069Q/H1069Q; w; initial diagnosis at age 18; LTX at age 18, liver cirrhosis, cholestasis, copper content: 1508 µg/g dry weight, no drug pretreatment |
| **D2** | 2 | f; T977M/T977M; initial diagnosis at age 19; LTX at age 19, liver cirrhosis, bile duct proliferation, mild adiposis, copper content: 1581 µg/g dry weight, no drug pretreatment |
| **D3** | 3 | f; V1262F/V1262F; initial diagnosis at age 14; LTX at age 20, active liver cirrhosis, pretreatment with D-Penicillamine (1200 mg/d for 6 years) to 3 months before LTX |
| **D4** | 4 | m; R778G/R778G; initial diagnosis at age 22; LTX at age 25, Liver cirrhosis with mild inflammation, zinc supplementation (zinc sulphate 250 mg/d for 13 years) |
| **D5** | **5** | m; R778G/R778G; decompensated liver cirrhosis, zinc supplementation |
| **D6** | **6** | m; H1069Q/H1069Q; decompensated liver cirrhosis, treatment with D- Penicillinamine (1500 mg/d for 33 years) |

Abbreviations used are: f, female; LTX, liver transplantation; m, male.
