# Supplementary material for: Laser ablation inductively coupled plasma mass spectrometry imaging of metals in experimental and clinical Wilson's disease
Source: J Cell Mol Med. 2015 Feb 20;19(4):806–14. doi: 10.1111/jcmm.12497 (PMC4395195; doi:10.1111/jcmm.12497)
Supplement: Supplementary file 9 [file jcmm0019-0806-sd9.doc]

**Supplementary Table 2**

Primers used in this study

| **Primers for mouse** | | |
| --- | --- | --- |
| **Gene** | **Acc. No.** | **Primer (5’→ 3’)** |
| mIL-1β | NM_008361.3 | for: TGT AAT GAA AGA CGG CAC ACC  rev: TCT TCT TTG GGT ATT GCT TGG |
| mTNF-α | NM_013693.2 | for: TCT TCT CAT TCC TGC TTG TGG  rev: GGT CTG GGC CAT AGA ACT GA |
| mNLRP3 | NM_145827.3 | for: CCC TTG GAG ACA CAG GAC TC  rev: GAG GCT GCA GTT GTC TAA TTC C |
| mASC | NM_023258.4 | for: GAG CAG CTG CAA ACG ACT AA  rev: GTC CAC AAA GTG TCC TGT TCT G |
| mTIMP-1 | AY622853 | for: TCC TCT TGT TGC TAT CAC TGA TAG CTT  rev: CGC TGG TAT AAG GTG GTC TCG TT |
| mMMP-9 | NM_013599 | for: CAG GAT AAA CTG TAT GGC TTC TGC  rev: GCC GAG TTG CCC CCA |
| mGAPDH | XM_001473623 | for: ACT GCC ACC CAG AAG ACT G  rev: CAC CAC CCT GTT GCT GTA G |
| **Primers for human** | | |
| **Gene** | **Acc. No.** | **Primer (5’→ 3’)** |
| hIL-1β | NM_000576 | for: TAC CTG TCC TGC GTG TTG AA  rev: TCT TTG GGT AAT TTT TGG GAT CT |
| hNLRP3 | AF410477 | for: GGA GAG ACC TTT ATG AGA AAG CAA  rev: GCT GTC TTC CTG GCA TAT CAC A |
| hATP7b | DQ015922 | for: TCA TCT TCT TTA TCT TGT GTA CCT TTG  rev: GAT TTG TAG GCC TGA ACG TAG AA |
| hCaspase-1 | BC062327 | for: CCG AAG GTG ATC ATC ATC CA  rev: ATA GCA TCA TCC TCA AAC TCT TCT G |
| hTNF-α | NM_000594 | for: GCT GCA CTT TGG AGT GAT CG  rev: GTT TGC TAC AAC ATG GGC ATC AG |
| hASC | AB023416 | for: AGT TTC ACA CCA GCC TGG AA  rev: TTT TCA AGC TGG CTT TTC GT |
| hTIMP-1 | NM_003254 | for: GGG CTT CAC CAA GAC CTA CA  rev: TGC AGG GGA TGG ATA AAC AG |
| hMMP-9 | NM_004994 | for: GAA CCA ATC TCA CCG ACA GG  rev: GCC ACC CGA GTG TAA CCA TA |
| hGAPDH | AF261085 | for: AGC CAC ATC GCT CAG ACA C  rev: GCC CAA TAC GAC CAA ATC C |
