# Supplementary material for: Laser ablation inductively coupled plasma mass spectrometry imaging of metals in experimental and clinical Wilson's disease
Source: J Cell Mol Med. 2015 Feb 20;19(4):806–14. doi: 10.1111/jcmm.12497 (PMC4395195; doi:10.1111/jcmm.12497)
Supplement: Supplementary file 10 [file jcmm0019-0806-sd10.doc]

**Supplementary Table 3**

Antibodies used in this study

| **Antibody** | **Cat. No.** | **Clonality*** | **Supplier** | **Species*** | **Dilution*** |
| --- | --- | --- | --- | --- | --- |
| **Primary antibodies** | | | | | |
| NLRP-3 | sc-66846 | Poly | Santa Cruz | h, m, r | 1:500 |
| mIL-1β/IL-1F2 | AF-401-NA | Poly | R&D Systems | m | 1:1000 |
| ATP7B | ab135571 | Poly | abcam | h,m | 1:250 |
| TIMP-1 | sc-5538 | Poly | Santa Cruz | h, m, r | 1:1000 |
| CASP-1 | ab108362 | Mono | abcam | h,m,r | 1:1000 |
| α-SMA | CBL171 | Mono | Cymbus | h,m,r | 1:1000 |
| TNF-R1 | sc-8436 | Mono | Santa Cruz | h, m, r | 1:1000 |
| MMP-9 | 3852S | Mono | Cell Signaling | h | 1:1000 |
| β-actin | A5441 | Mono | Sigma-Aldrich | h, m, r | 1:10000 |
| **Secondary antibodies** | | | | | |
| IgG-HRP | sc-2004 | N/A | Santa Cruz | r | 1:5000 |
| IgG-HRP | sc-2005 | N/A | Santa Cruz | m | 1:5000 |
| IgG-HRP | sc-2056 | N/A | Santa Cruz | g | 1:5000 |

* Abbreviations used are: Mono, monoclonal antibody; Poly, polyclonal antibody; N/A, not applicable; h = human, m = mouse, r = rat, g =goat.
